# Calcimimetic R-568 vasodilatory effect on mesenteric vascular beds from normotensive (WKY) and spontaneously hypertensive (SHR) rats. Potential involvement of vascular smooth muscle cells (vSMCs)

**DOI:** 10.1371/journal.pone.0202354

**Published:** 2018-08-09

**Authors:** Natalia Di Pietro, Maria Assunta Potenza, Sara Di Silvestre, Francesco Addabbo, Nadia Di Pietrantonio, Pamela Di Tomo, Caterina Pipino, Domitilla Mandatori, Carola Palmerini, Paola Failli, Mario Bonomini, Monica Montagnani, Assunta Pandolfi

**Affiliations:** 1 Department of Medicine and Aging Sciences, University “G. d’Annunzio” of Chieti-Pescara, Chieti, Italy; 2 Aging and Translational Medicine Research Center (CeSI-MeT), University “G. d’Annunzio” of Chieti-Pescara, Chieti, Italy; 3 “G. d’Annunzio” University Foundation, Chieti, Italy; 4 Department of Pharmacology and Human Physiology, University of Bari, Bari, Italy; 5 Department of Medical, Oral and Biotechnological Sciences, University “G. d’Annunzio” of Chieti-Pescara, Chieti, Italy; 6 Department of Neurofarba, Pharmacology and Toxicology Unit, University of Florence, Florence, Italy; Universidade Federal do Rio de Janeiro, BRAZIL

## Abstract

The potential role of calcimimetics as vasculotropic agents has been suggested since the discovery that calcium sensing receptors (CaSRs) are expressed in cardiovascular tissues. However, whether this effect is CaSR-dependent or -independent is still unclear. In the present study the vascular activity of calcimimetic R-568 was investigated in mesenteric vascular beds (MVBs) isolated from Spontaneously Hypertensive rats (SHR) and the relative age-matched Wistar-Kyoto (WKY) control rats. Pre-constricted MBVs were perfused with increasing concentrations of R-568 (10 nM– 30 μM) resulting in a rapid dose-dependent vasodilatation. However, in MVBs from SHR this was preceded by a small but significant vasoconstriction at lowest nanomolar concentrations used (10–300 nM). Pre-treatment with pharmacological inhibitors of nitric oxide (NO) synthase (NOS, L-NAME), K_Ca_ channels (CTX), cyclo-oxygenase (INDO) and CaSR (Calhex) or the endothelium removal suggest that NO, CaSR and the endothelium itself contribute to the R-568 vasodilatory/vasoconstrictor effects observed respectively in WKY/SHR MVBs. Conversely, the vasodilatory effects resulted by highest R-568 concentration were independent of these factors. Then, the ability of lower R-568 doses (0.1–1 μM) to activate endothelial-NOS (eNOS) pathway in MVBs homogenates was evaluated. The Akt and eNOS phosphorylation levels resulted increased in WKY homogenates and Calhex significantly blocked this effect. Notably, this did not occur in the SHR. Similarly, vascular smooth muscle cells (vSMCs) stimulation with lower R-568 doses resulted in Akt activation and increased NO production in WKY but not in SHR cells. Interestingly, in these cells this was associated with the absence of the biologically active dimeric form of the CaSR thus potentially contributing to explain the impaired vasorelaxant effect observed in response to R-568 in MVB from SHR compared to WKY. Overall, these findings provide new insight on the mechanisms of action of the calcimimetic R-568 in modulating vascular tone both in physiological and pathological conditions such as hypertension.

## Introduction

The extracellular calcium (Ca^2+^) sensing receptors (CaSRs) are important in maintaining and regulating mineral ion homeostasis in parathyroid, kidney, gut and bone tissues [1, 2]. The discovery that CaSRs are widely expressed in the heart as well as in the blood vessels has suggested their physiological involvement in cardiovascular processes such as vessel remodeling and calcification; similarly, acute CaSR-mediated activation has been proposed to participate in the maintenance of vascular tone and modulation of blood pressure levels [3–8]. However, whether the aforementioned cardiovascular effects are secondary to CaSR-dependent regulation of Ca^2+^ homeostasis in other organs or directly related to CaSR modulation in vascular tissues is still unclear, and the exact mechanisms underlying CaSR activation remain to be elucidated [3].

In the local control of vascular tone, the role played by the endothelial-mediated release of the vasodilator nitric oxide (NO) is universally recognized [9]. A direct vascular function of the endothelial CaSR is supported by the finding that stimulation of human aortic endothelial cells with polyamine spermine (CaSR type I agonist) leads to an increase of intracellular Ca^2+^ accompained by NO production [10], whereas treatment with the CaSR antagonist NPS2390 attenuates extracellular Ca^2+^-dependent NO-mediated relaxation in endothelium-intact aortic rings from mice [11].

Interestingly, CaSR seems also actively involved in the modulation of myogenic tone in response to changes in Ca^2+^ concentration, as initially demonstrated in rat subcutaneous small arteries and more recently observed in aorta and mesenteric artery of transgenic mice with specific CaSR knockout (CaSR KO) in vascular smooth muscle cells (vSMCs) [5, 12]. These data, together with other recent evidences, suggest that CaSR may mediate a dual direct activity on blood vessels: activation of endothelial CaSR results in a NO-dependent pro-relaxing effect, whereas stimulation of CaSR located on vSMCs may elicit a pro-contractile effect [13, 14].

Based on these observations, the development and investigation of compounds able to modulate CaSR activity might represent an additional strategy in the control of cardiovascular homeostasis. Nevertheless, *in vitro* and *ex vivo* studies on vSMCs and endothelial cells suggest that drugs proposed as calcimimetics might be able to regulate vascular tone independently from CaSR activation [15–18]. For example, a CaSR-independent relaxant effect of calcimimetics has been ascribed to their ability to inhibit the Ca^2+^ influx via L-type Ca^2+^ channels [15, 16]. Other authors have proposed that calcimimetic-mediated NO production might depend on stimulation of the big or large conductance Ca^2+-^activated potassium channel (BKCa) channels in vSMCs [18], while activation of intermediate conductance Ca^2+^-activated potassium (IKCa) channels in endothelium may explain vasorelaxation through release of endothelium-derived hyperpolarizing factor (EDHF) [19, 20].

These observations are in line with our recent findings [17], evaluating the activity of calcimimetic R-568, a type II indirect allosteric CaSR modulator [21], in human umbilical vein endothelial cells (HUVECs). We found that, although expressed in endothelial cells, CaSR protein was mainly distributed in cytoplasm, while the functional CaSR dimers usually localize on the plasma membrane. In addition, increased intracellular Ca^2+^ concentration and endothelial NO synthase (eNOS) activation in response to both R-568 and its enantiomer S-568 were unchanged in the absence or the presence of the CaSR blocker Calhex-231 [17]. Accordingly, an acute and CaSR-independent hypotensive effects has been reported following infusion of R-568 and S-568 in rat renal and mesenteric arteries [22]. Therefore, whether R-568 may exert CaSR-dependent or CaSR-independent vascular effects that might physiologically contribute to regulation of vascular tone is still under debate.

Finally, to add complexity, it is important to consider that calcimimetic-dependent vascular effects might vary under conditions altering endothelial function/integrity such as hypertension. On this regard results are still controversial, since some studies report an *in vivo* calcimimetic hypotensive effects in both normotensive [23] and spontaneously hypertensive (SHR) rats [24] while others found that R-568 significantly decreased blood pressure in uremic and SHR animals, but not in normotensive rats [25]. Importantly, when these drugs are administered *in vivo*, the hypothetical contribution of direct vascular effects and the calcimimetic-mediated suppression of parathyroid hypertensive factors are hard to dissect [3].

This last observation suggests that studies on isolated and perfused vessels might offer the possibility to evaluate the direct vascular effects of calcimimetics avoiding confounding factors such as modulation of calcium in non-cardiovascular districts. In the present study, the potential CaSR-dependent/independent vasodilatory effects of R-568, along with the intracellular signaling pathway activated, was comparatively evaluated in mesenteric vascular bed (MVB) and cultured vSMCs, from SHR rats and normotensive WKY controls.

## Materials and methods

### Animals

All procedures in animals were performed in accordance with the Guide for the Care and Use of Laboratory Animals (NIH Publication No. 85–23, revised 1996) and approved by the Committee on the Ethics of Animal Experiments of the University of Bari (Prot. N. 60901-X/10) under Authorization for the Use of Laboratory Animals (Italian Government, Ministry of Health, Prot. N.86851493).

Twenty 8-week old male Spontaneously Hypertensive rats (SHR, 311.0 ± 7.14 g) and twenty age-matched normotensive Wistar-Kyoto (WKY, 309.5 ± 9.7 g) control rats were obtained from Charles River (Milan, Italy) and used in all studies. Animals were housed in 12 hours light/dark cycle, at 22°C, and allowed *ad libitum* access to diet and water.

The blood pressure of WKY and SHR rats was randomly measured in conscious rats using BP-2000 (Visitech Systems, Apex, North Carolina, USA), a non-invasive computerized system for recording blood pressure from rodent tails. Systolic blood pressure, measured at 8 weeks, ranged from 119 to 137 mmHg in WKY and from 153 to 176 mmHg in SHR.

Rats were anesthetized with sodium pentobarbital (80 mg/kg body weight i.p.), heparinized (400 U.I./100g body weight i.p.) and euthanized by cervical dislocation. All efforts were made to minimize animal suffering.

### Reagents

Heparin was purchased from Pfizer (Pfizer Srl, Rome, Italy); noradrenaline (NA), acetylcholine (ACh), Nω-nitro-L arginine methyl ester (L-NAME), charybdotoxin (CTX) and indomethacin (INDO) were purchased from Sigma (Sigma-Aldrich Chemicals, St.Louis, MO, USA). Stock solutions of NA (100 mM) and ACh (10 mM) were prepared with distilled water. R-568 hydrochloride powder was provided by Amgen (Amgen, Inc., Thousand Oaks, CA, USA), resuspended in distilled water at 2 mM concentration and stored at -20°C. Calhex-231 (Santa Cruz Biotechnology, Heidelberg, Germany) was resuspended in ethanol at 10 mM concentration and stored at -20°C. Final dilutions of all drugs were prepared in modified Krebs-Henseleit solution immediately before use.

### *Ex-vivo* vascular studies

Mesenteric vascular beds (MVB) were isolated and removed from fourteen WKY and fourteen SHR rats as previously described [26]. Briefly, MVB mounted in a temperature-controlled moist chamber (type 834/1, Hugo Sachs Elektronik, March-Hungstetten, Germany) were perfused with modified Krebs-Henseleit solution continuously gassed with a mixture of 95% O_2_ and 5% CO_2_ (pH 7.4). A constant flow rate of 5 ml/min through the MVB was maintained using a peristaltic pump (ISM 833; Hugo Sachs Elektronik, March-Hungstetten, Germany). Drug solutions were infused into the perfusate proximal to the arterial cannula using another peristaltic pump. After an equilibration period (30–40 min), changes in perfusion pressure (PP) were measured with a Pressure Transducer System (SP 844 Capto, Horten, Norway) and recorded continuously using data acquisition and analysis equipment (PowerLab System, ADInstruments, Castle Hill, Australia).

Vasodilator responses in MVB were measured in vessels pre-constricted with NA according to previous studies [27], to obtain comparable baseline PP values of approximately 120 mmHg in WKY and SHR, MVB preparations were continuously perfused with NA 10 and 3 μM, respectively. Dose-response curves measuring vasodilation (decrease in PP) in response to R-568 were obtained by adding increasing concentrations of R-568 (10 nM– 30 μM/4 min perfusion) into the perfusate. Relative changes in PP at steady-state reached with each dose were measured and expressed in mmHg. For all vasodilation experiments, data from each curve were then normalized to PP obtained with a maximally stimulating dose of ACh (1 μM, 100% representing initial steady-state perfusion pressure and 0% representing maximal reduction in response to ACh). In some experiments, R-568-induced relaxation was measured after 20-min treatment with NOS inhibitor L-NAME (100 μM), alone or combined with large and intermediate-conductance Ca^2+^-activated potassium channels (BK_Ca_ and IK_Ca_) antagonist CTX (300 nM) and cyclooxygenase 1/2 (COX1/2) inhibitor INDO (10 μM). In other experiments, R-568-induced relaxation was measured in the absence and in the presence of CaSR blocker Calhex-231 (3 μM), or in the absence of endothelium. Endothelium removal was performed by flushing vessels intermittently with air for several minutes as previously described [27] and verified by complete lack of vasodilation induced by infusion of 1 μM ACh.

### Cell cultures

Vascular smooth muscle cells (vSMCs) were obtained from thoracic aortas of six normotensive (WKY-vSMCs) and six hypertensive (SHR-vSMCs) animals as previously described [28–30].

Briefly, thoracic aortas were dissected, transferred into a Petri dish containing Dulbecco's Modified Eagle’s Medium low glucose (DMEM-LG) and discharged from fatty tissues. Subsequently, aortas were cut longitudinally and endothelial cells were removed through rubbing. The three vessel layers were cut into small pieces (1–2 mm) and maintained for 2 hours at 37°C in DMEM-LG containing 0.1% elastase and 0.1% collagenase type IA. Following a centrifugation to 250 g for 10 min, the supernatant were discarded and the cells were re-suspended in culture medium composed of Ham's F12 Medium (Sigma-Aldrich Chemicals, St.Louis, MO, USA), 20% Fetal Bovine Serum (FBS, Gibco-Life Technologies, Monza, Italy), 1% penicillin/streptomycin and 1% L-glutamine (Sigma-Aldrich Chemicals, St.Louis, MO, USA). The cells were growth under a controlled atmosphere (5% CO2 and 37°C) until confluence (8 ± 10 days). The characterization of α- smooth muscle actin (α-SMA) expression in WKY/SHR-vSMCs was performed by flow cytometry.

In all experiments, vSMCs were starved for 16 hours with Ham's F12 Medium (Sigma-Aldrich Chemicals, St.Louis, MO, USA) containing 0.1% Fetal Bovine Serum (FBS, Gibco-Life Technologies, Monza, Italy), and subsequently stimulated with R-568 molecule (1 μM). In some experiments, before stimulation with R-568, cells were pre-incubated for 15 minutes with CaSR Inhibitor, Calhex 231 (1 μM).

### Flow cytometry

The protein expression of α-SMA in permeabilized WKY- and SHR- vSMCs was evaluated by fluorescent-activated cell sorting (FACS) FACS Canto II (BD Bioscences, California, USA).

Briefly, WKY- and SHR- vSMCs were pelleted by centrifugation at 1500 rpm for 5 min and resuspended in PBS. Then, 5×10^5^ cells/sample were fixed and permeabilized using FACS Lysing and Permeabilizing Solution (BD, CAT. 349202 and 340973 respectively). Permeabilized cells were incubated with a mouse monoclonal against α-SMA (Sigma-Aldrich, 1:100) for 30 minutes on ice and successively stained with the corresponding secondary antibody (Anti-mouse FITC, 1:100, Jackson ImmunoResearch) for 30 min on ice. To assess non‐specific fluorescence, samples were stained with the secondary antibody alone (control).

### NOS activity

NOS activity was evaluated in the WKY and SHR cells by measuring the conversion of L-[3H]-arginine into L-[3H]-citrulline as previously described [28, 31]. Briefly, WKY- and SHR-vSMCs were grown at confluence and then stimulated with R-568 (1 μM) in the absence or the presence of Calhex-231(1 μM). Subsequently, cells were detached, resuspended in 0.2 ml of reaction buffer (20 mmol/L Hepes-Na^+^, 0.5 mmol/L EDTA, 1 mmol/L dithiothreitol, pH 7.2) and sonicated on ice. In each test tube containing 100 μl of cell lysate, the following reagents were added: 2 mmol/L NADPH, 1.5 mmol/L CaCl_2_, 0.1 mmol/L tetrahydrobiopterin (BH4, Sigma-Aldrich Chemicals, St.Louis, MO, USA), 2.5 μCi L-[3H]-arginine (corresponding to 0.4 μM) (Perkin Elmer SpA, Milan, Italy). After 15 min incubation at 37°C, the reaction was stopped by adding 2 ml Hepes-Na^+^ pH 6 containing 2 mmol/L EDTA; the entire reaction mixture was applied to 2 ml of Dowex AGWX8-200 columns (Sigma-Aldrich Chemicals, St.Louis, MO, USA) and eluted with 4 ml of water. The radioactivity corresponding to L-[3H]-citrulline content in the eluate was measured by a liquid scintillation analyzer (Perkin Elmer SpA, Milan, Italy). NOS activity was expressed as pmol/NO/min/mg protein total.

### Immunoblotting analysis

Expression of protein of interest in vSMCs and whole vessel lysates was assessed by immunoblotting with specific antibodies according to standard methods [32]. In each sample, total protein content was quantified using the BCA Protein Assay Kit (Pierce Biotechnology Inc, Rockford, USA). Samples were subjected to electrophoretic separation by 8–10% SDS-PAGE. For tissue samples, MVB homogenates (35 μg) were immunoblotted with α-eNOS (BD Transduction Laboratories, CA, USA), phospho-eNOS^Ser1179^, Akt, phospho-Akt^Ser473^ (Cell Signaling Technology, Leiden, Netherlands), or monoclonal anti-α smooth muscle actin (α-actin, Sigma-Aldrich Chemicals, St.Louis, MO, USA) antibodies (1:1000, at 4° C overnight). For vSMCs, lysates (30 μg) were immunoblotted with CaSR (Thermo Fisher Scientific, Milan, Italy) and its positive (HEK293 CaSR transiently transfected cell lysate) and negative (HEK293 empty vector transfected control cell lysate) controls (both from Novus Biologicals, UK), Akt, phospho-Akt^Ser473^ and β-actin (Sigma-Aldrich Chemicals, St.Louis, MO, USA) antibodies (1:1000, at 4° C overnight).

Incubation with specific mouse or rabbit HRP-linked secondary antibodies (1:3000; Santa Cruz Biotechnology, Heidelberg, Germany) was performed for 1 h at room temperature. Immunoblotting results were visualized using enhanced chemiluminescence (ECL) reagents (Amersham Pharmacia Biotech SpA, Milan, Italy) by Molecular Imager^®^ ChemiDoc^TM^ XRS System (Bio-Rad Laboratories Srl, Milan, Italy). Images were captured with QuantityOne Software (Bio-Rad Laboratories Srl, Milan, Italy) and blots quantified by scanning densitometry (ImageJ, NIH, Bethesda). In some experiments, the immunoreactive bands were detected using the Alliance 4.7 system (Uvitec Limited, Cambridge, UK).

### Statistical analysis

Results were expressed as mean ± SEM of *n* experiments (*n =* number of rats) for *ex-vivo* experiments, while *in-vitro* results were expressed as mean ± SD. Two-way ANOVA for repeated measures and Student’s *t* tests (paired or unpaired) were used as appropriate. Values of p < 0.05 were considered to indicate statistical significance.

## Results and discussion

### Vascular effects of R-568 in MVB from WKY and SHR rats

Calcimimetics are allosteric CaSR activators proposed as potential anti-hypertensive drugs following the discovery that their systemic administration reduces blood pressure in animal models [3–5]. However, although CaSRs are expressed on endothelial and vSMC cells, their role in the regulation of vascular tone under healthy and pathophysiological conditions has not been definitively clarified [33]. Moreover, both CaSR-dependent and CaSR–independent mechanisms have been proposed to explain calcimimetic hemodynamic effects with uncertain and sometimes controversial results [5, 10, 12, 15–18].

To shed light on these issues, we investigated cellular mediators and signaling mechanisms responsible for the direct vascular effects of the calcimimetic R-568 on whole perfused vessels and on isolated vSMCs obtained from spontaneously hypertensive rats (SHR) and their relative Wistar-Kyoto (WKY) controls. SHR animals share many common features with human genetic hypertension and, at 8-week of age, they manifest endothelial dysfunction and are prone to vascular dysreactivity [34]. Importantly, changes in endothelial function observed in mesenteric vascular bed (MVB) have been shown to parallel, or even precede, changes in vascular reactivity, elasticity, and volume of large conductance arteries [27]; therefore, the behaviour of this vascular district is considered a useful proxy for the function of vascular beds that contribute more directly to systemic blood pressure [27, 33].

MVB from SHR rats are significantly more sensitive and responsive to the vasoconstrictor actions of NA with respect to MVB from WKY [27]. Based on this, and according to previous experience [26, 27, 35, 36] we infused NA at 3 μM or 10 μM concentration, respectively, to obtain a similar baseline perfusion pressure (PP) of ~120 mmHg, rather than infusing an identical NA dose for both SHR and WKY. The NA concentration chosen was the dose achieving submaximal vasoconstriction in SHR and WKY, respectively, from dose-response curves (10 nm—10 μM) preliminary obtained ex-vivo. Before administration of R-568, a supramaximal dose of the vasodilator ACh (1 μM) was added to verify endothelial integrity. Subsequently, increasing concentrations of R-568 (10 nM– 30 μM/4 min perfusion) were infused in a non-cumulative manner into the preparations.

**Fig 1:**
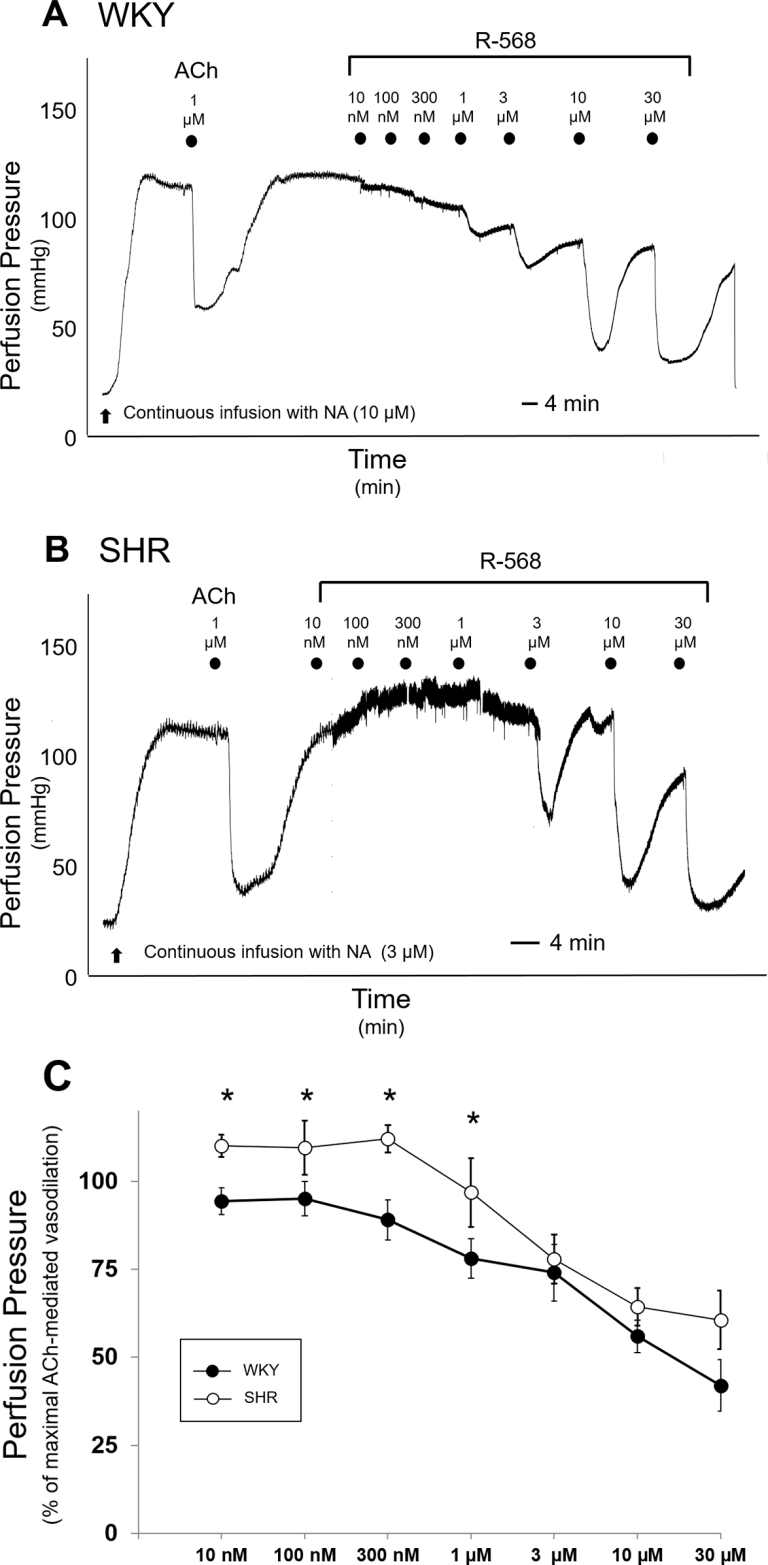
Vascular effects of R-568 in MVB from WKY and SHR rats. Mesenteric vascular beds (MVB) from 8-week old WKY and SHR rats were isolated and prepared as described in Methods. PP at approximately 120 mmHg was maintained by continuous infusion with NA (10 μM and 3 μM in WKY and SHR rats, respectively). (A) and (B) Representative tracings of vasodilator responses to submaximal ACh dose (1 μM) and to increasing concentrations of R-568 (10 nM—30 μM) in WKY (A) and SHR (B) are shown. Each symbol represents the start of a 4-min perfusion. (C) Dose-response curves for R-568-induced vascular effects were obtained from MVB of WKY and SHR. Results are the mean ± SEM of 7 (WKY) and 6 (SHR) independent experiments. Data from each curve were normalized by defining 100% as the initial steady state PP and 0% as the maximal reduction in PP obtained in WKY treated with 1 μM ACh. *p < 0.01 *vs* respective values.

As shown in Fig 1A, administration of R-568 in MVB from WKY resulted in a rapid, reversible, and dose-dependent reduction of PP, starting with the lowest concentration used (10 nM). Maximal effect was observed at 30 μM concentration (Fig 1A) (p < 0.01 *vs* WKY PP values at initial steady state). With respect to WKY, vascular activity of R-568 on MVB from SHR was noticeably different. Indeed, as shown in Fig 1B, the vasorelaxant responses to R-568 became visible at concentrations higher than 1 μM (3–30 μM); concomitantly, the lower nM concentrations of R-568 (10–300 nM), including the 1 μM concentration, evoked a slight but significant increase of PP (Fig 1C; p < 0.05 *vs* SHR PP at initial steady state).

Thus, vascular effects of R-568 in MVB from SHR did not completely overlap with effects observed in MVB from WKY (Fig 1C; p < 0.01 *vs* each respective WKY values): the high doses of R-568 induced a comparable vasodilation in both strains whereas, in the lower range of concentrations, stimulation with R-568 elicited vasodilation in WKY and vasoconstriction in SHR.

### Evaluation of endothelial mediators involved in vascular effects of R-568

**Fig 2:**
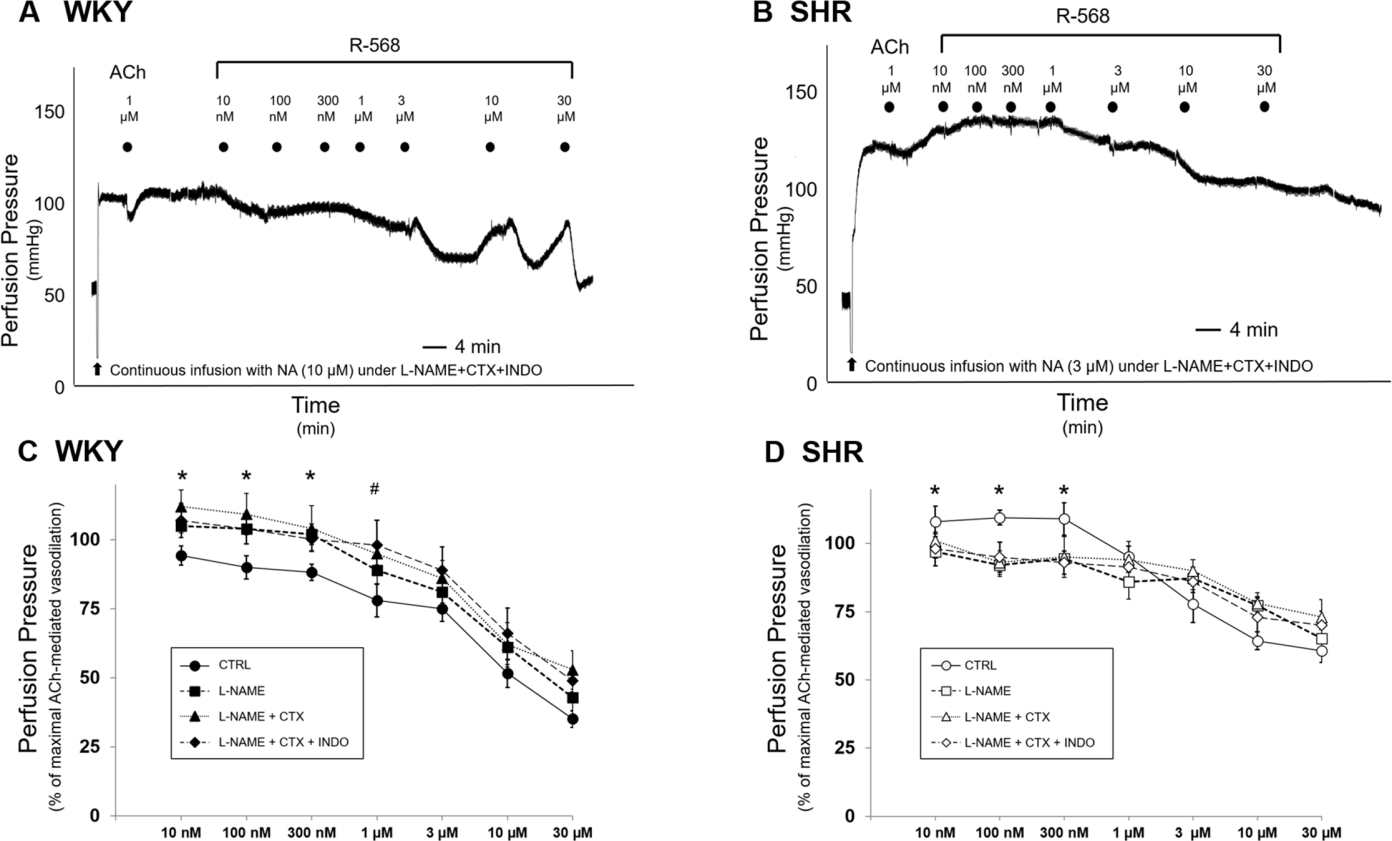
Evaluation of endothelial mediators involved in vascular effects of R-568 in MVB from WKY and SHR rats. (A) and (B) Representative tracings of vascular response to R-568 (10 nM—30 μM) in MVB from WKY (A) or SHR (B) pretreated with L-NAME, CTX and INDO are shown. Each symbol represents the start of a 4-min perfusion. (C) MVB isolated from 8-week old WKY (closed symbols) were stimulated with increasing concentrations of R-568 in the absence (circles) or in the presence of L-NAME (100 μM/20 min; squares), L-NAME+CTX (300 nM/20 min; triangles), L-NAME+CTX+INDO (10 μM/20 min; diamonds). Results are the mean ± SEM of 4 WKY independent experiments. (D) MVB isolated from 8-week old SHR (open symbols) were stimulated with increasing concentrations of R-568 in the absence (circles) or in the presence of L-NAME (100 μM/20 min; squares), L-NAME+CTX (300 nM/20 min; triangles), L-NAME+CTX+INDO (10 μ M/20 min; diamonds). Results are the mean ± SEM of 4 WKY and 4 SHR independent experiments. *p < 0.04 and ^#^p < 0.05 *vs* respective CTRL curve.

Nitric oxide (NO), BK_Ca_ and IK_Ca_ channels, and arachidonic acid metabolites are all involved in the control of vessel tone. To analyze the potential contribution of these vascular mediators on R-568-induced vascular effects, dose-response curves to R-568 were repeated in MVB pretreated with pharmacological inhibitors of NO synthase (L-NAME), K_Ca_ channels (CTX), and cyclo-oxygenase (INDO) (Fig 2).

In WKY, as expected, pretreatment with L-NAME (100 μM/20 min) significantly blunted vasorelaxation to ACh. Interestingly, L-NAME pretreatment abrogated vasodilation in response to the lowest concentrations of R-568 (10–300 nM; Fig 2C; p < 0.05 *vs* CTRL curve), but did not significantly influence vasodilation induced by the highest concentrations of R-568 (Fig 2C).

To evaluate the involvement of additional endothelial factors contributing to R-568-induced vasodilation, MVB were subsequently treated with CTX (300 nM/20 min) and INDO (10 μM/20 min) in combination with L-NAME. Under these conditions, residual ACh-mediated vasodilation was negligible (Fig 2A). When treatment with L-NAME was combined with CTX and INDO, a further inhibitory effect was observed on vasodilation induced by R-568 at 1 μM concentration (Fig 2A and 2C; p < 0.05 *vs* CTRL curve). Conversely, in the higher micromolar range, vasodilatory effects to R-568 were not significantly reduced by the combined treatment with all inhibitors together (Fig 2A and 2C).

In SHR, similar to WKY, pretreatment with L-NAME did not significantly reduce the vasodilator responses obtained with the higher doses of R-568 (Fig 2D). The combined treatment with L-NAME, CTX and INDO was unable to block the R-568-mediated vasodilation as well (Fig 2D). Surprisingly enough, though, the small vasoconstrictor effect evoked by low doses of R-568 in SHR was completely blocked when MVB was incubated with L-NAME, alone or in combination with CTX and INDO (p < 0.05 *vs* CTRL curve) (Fig 2B and 2D).

### Effect of endothelium removal and CaSR inhibition on vascular response to R-568

The behavior of R-568 in vessels pretreated with L-NAME, CTX and INDO suggests that, for the higher concentrations tested, vasodilation obtained with R-568 might not require endothelial contribution. On the other hand, at concentrations lower than 1 μM, effects produced by R-568 were quite different in MVB from WKY (where it induced vasodilation) with respect to SHR (where it propended to vasoconstriction).

**Fig 3:**
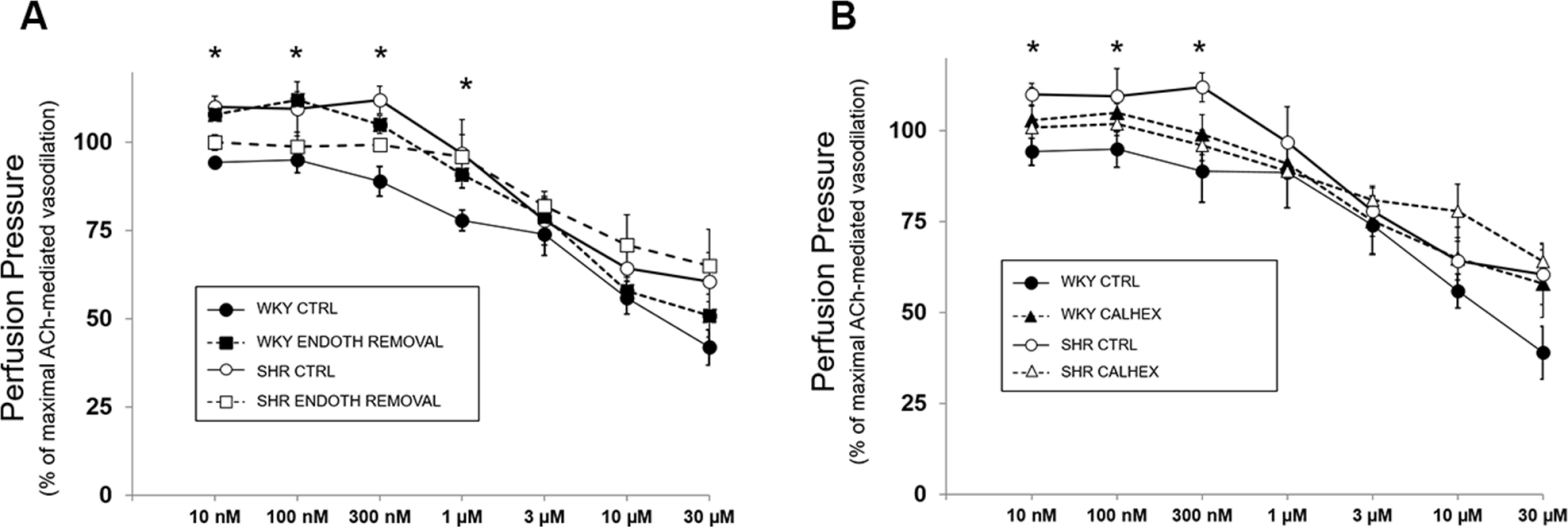
Effect of endothelium removal and CaSR inhibition on vascular response to R-568. (A) MVB isolated from 8-week old WKY (closed symbols) and SHR (open symbols) were stimulated with increasing concentrations of R-568 in the presence (circles) or in the absence (squares) of endothelium. Results shown are the mean ± SEM of 3 (WKY) and 3 (SHR) independent experiments. Data from each curve were normalized as in Fig 1. (B) MVB isolated from 8-week old WKY (closed symbols) and SHR (open symbols) were stimulated with increasing concentrations of R-568 in the absence (circles) or after pretreatment with (diamonds) Calhex-231 (3 μM/20 min). Results are the mean ± SEM of 3 WKY and 3 SHR independent experiments. Data from each curve were normalized as in Fig 1. *p < 0.05 *vs* respective CTRL curve.

To unquestionably ascertain the involvement of endothelium in R-568-mediated effects, dose-response curves to R-568 were repeated in both WKY and SHR MVB before and after endothelial removal, this last condition verified by complete lack of ACh-mediated vasodilation (data not shown). In WKY rats, consistent with results obtained under L-NAME, CTX and INDO treatment, endothelium removal inhibited R-568-mediated vasodilation in the range of 10 nM—1 μM, but did not abrogate vasodilation at R-568 concentrations higher than 1 μM (Fig 3A; p < 0.05 *vs* respective CTRL curve). Likewise, in SHR rats, endothelium removal overlapped effects observed under L-NAME pretreatment: vasodilation in response to high concentrations of R-568 remained unchanged in the presence or in the absence of endothelium (Fig 3A); once again, as described for L-NAME, vasoconstriction to lower concentrations of R-568 was not visible in vessels without endothelium (Fig 3A; p < 0.05 *vs* respective CTRL curve).

The next obligatory step was to verify the possibility that vascular effects in response to R-568 would indeed be mediated by CaSR activation. Thus, in another set of experiments, the dose-response curves to R-568 were compared in the absence and in the presence of CaSR blocker Calhex-231 (3 μM/20 min) in MVB from WKY and SHR rats. As expected, ACh-mediated vasodilation was not modified by pretreatment with Calhex-231 (data not shown). Fig 3B demonstrates that, similar to endothelium removal, inhibition of CaSR did not significantly reduce vasodilation to high doses of R-568 in both SHR and WKY preparations On the other hand, vasoactive effects of R-568 in the 10 nM– 1 μM concentration range were significantly reduced under CaSR inhibition, in WKY as well as in SHR (Fig 3B). Interestingly, blockade of CaSR was able to abrogate both the vasodilation in MVB from WKY as well as the vasoconstriction observed in MVB from SHR (p < 0.05 *vs* respective CTRL curve).

The CaSR-independent vasodilation observed in response to high doses of R-568 in our study is consistent with results obtained with other calcimimetics: for example, the arterial relaxation induced by both cinacalcet and calindol was not substantiallly blocked by the CaRS inhibitor Calhex 231 in rat mesenteric vessels [16], suggesting the involvement of alternative/additional mechanisms. Similarly, at doses exceeding 70 μM, infusion of both R-568 and its enantiomer S-568 in rat mesenteric and renal arteries has been shown to equally increase blood flow [22]. Given the largely stereospecific activity of calcimimetics, the Authors concluded that the comparable vasodilation obtained with both R-568 and S-568 was unlikely mediated by CaSR activation [22]. One alternative explanation for the off-target effects of calcimimetics is based on their chemical structure: since these molecules are phenylalkylamine derivatives, their ability to modulate vascular relaxation and blood pressure has been mainly ascribed to potential interaction on L-type Ca^2+^ channels [16, 33]. Although, at present we cannot provide any direct evidence of an inhibitory effect of R-568 on L-type Ca^2+^ channels, this may help to comprehend why, in our study, vasodilation obtained with high doses of R-568 did not change in the absence or presence of endothelium, and was not significantly different between SHR and WKY.

Similarly, we cannot exclude that hyperpolarization might have a role in mediating R-568 comparable vasodilator effects in SHR and WKY, in the high range of concentrations tested: treatment with CTX, which blocks K_Ca_ channels in a nonselective fashion [37], might not affect small conductance Ca^2+^-activated potassium channel (SK_Ca_), voltage-gated potassium channel Kv1.3, or KATP channels. Thus, especially in MVB, where hyperpolarization contributes to the vasodilator response of several agonists [38–41], it remains possible that part of the off-target vasodilation by R-568 might involve hyperpolarization-mediated mechanisms.

In contrast to the result obtained at high R-568 concentration, the vasodilatory effect observed at low doses of R-568 in MVB from WKY was mediated by CaSR and dependent on endothelial factors, in agreement with other studies demonstrating the necessary role of CaSR and endothelium in the hypotensive effects of calcimimetics [12, 23, 42]. Conversely, in MVB from SHR rats, the lowest concentrations of R-568 evoked a dose-dependent vasoconstrictive effect that was both endothelium- and CaSR-mediated. This was somewhat surprising because, if any, the impaired endothelial function in vessels from SHR was expected to reduce the vasodilatory ability of R-568. Although puzzling, these findings should take into account the genetic background of SHR that may affect endothelial production of vasoactive mediators, resulting in abnormal/unbalanced vascular responsiveness [43]. For example, uncoupling of eNOS is known to generate reactive oxygen species (ROS) instead of NO [44, 45]. In this case, phosphorylation and activation of endothelial eNOS by R-568 may increase ROS levels and elicit subsequent vasoconstriction. Preliminary immunohistochemical experiments in our study suggest that levels of nitrotyrosine (indicating peroxinitrite production) in response to R-568 were higher in aortas isolated from SHR when compared with samples from WKY; on the same line, in immunofluorescence experiments, nuclear red fluorescence to dihydroethidium DHE (indicative of O^**2-**^ production) in response to R-568 was higher in aortic rings from SHR with respect to aortic rings from WKY (data not shown). Thus, in vessels from SHR, L-NAME pre-treatment or endothelial removal might mitigate the vasoconstriction elicited by R-568-mediated stimulation of uncoupled eNOS. This hypothesis is supported by data showing that treatment with the ROS scavenger Tempol was able to block the age-related development of high blood pressure in hypertensive rats [46]. On the same line, treatment with the antioxidant N-acetylcisteine (NAC) has been shown to effectively reduce blood pressure in young (5 weeks of age) SHR rats [47], underscoring the role played by ROS in the early stages of hypertension development. Alternatively, activation of CaSR might be involved in several signaling cascades related to production of either vasoconstrictor or vasodilator factors. Indeed, in the vascular district, CaSR activation has been associated with vasoconstriction and signalling via mitogen-activated protein kinases [48–50]. Thus, it is plausible that in vessels from SHR stimulation with R-568 may unmask vasoconstrictive effects not adequately counterbalanced by endothelial impaired production of NO. Although our present findings are too preliminary to exclude or confirm any hypothesis, these observations underline the specific behavior of R-568 under hypertensive conditions.

### Activation of signaling pathways by R-568 in MVB from WKY and SHR

**Fig 4:**
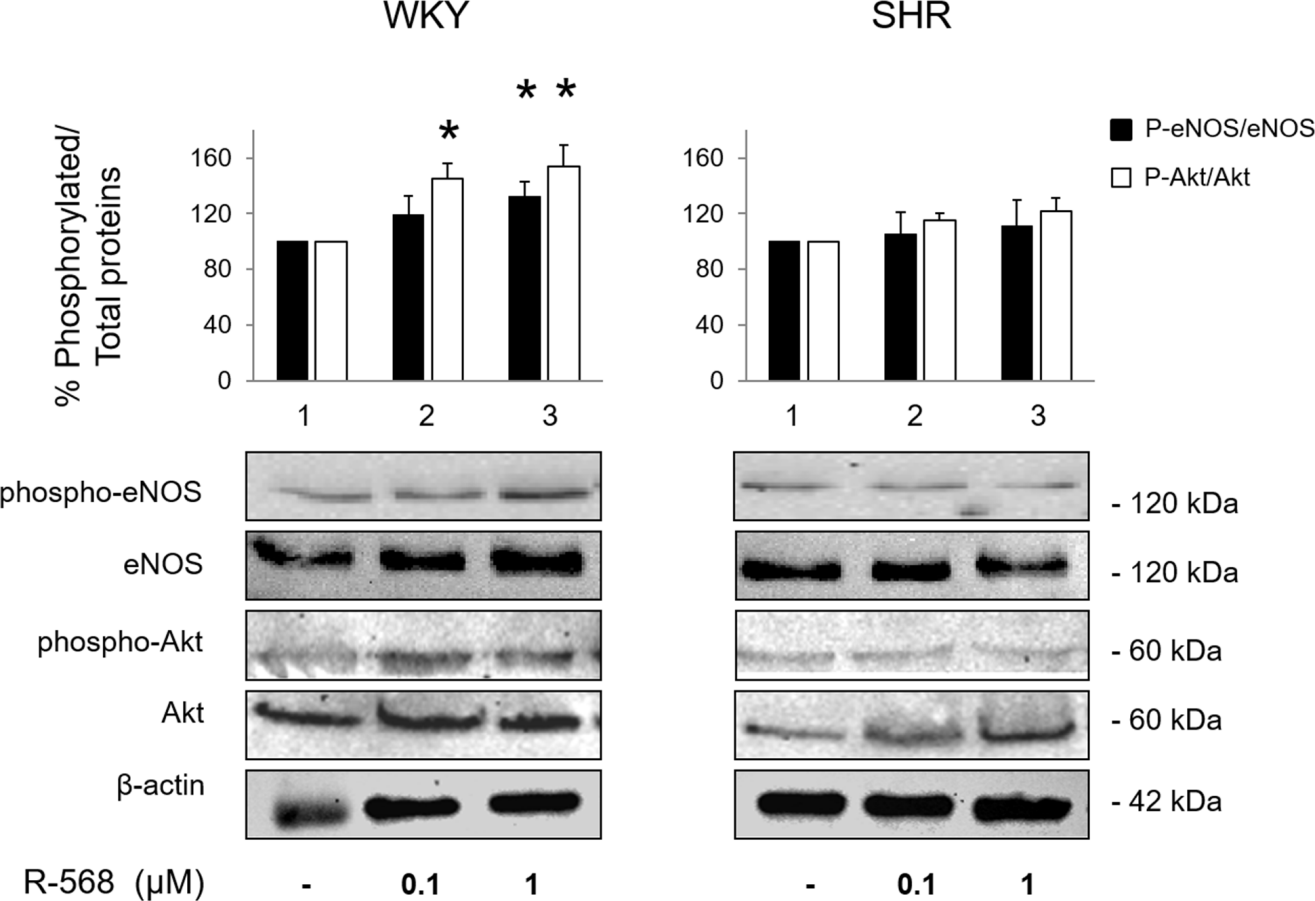
Evaluation of signaling pathways activated by R-568 in MVB from WKY and SHR. MVB homogenates from experiments described in Fig 1 were subjected to immunoblotting for total and phosphorylated forms of Akt (phospho-Akt^Ser473^), and eNOS (phospho-eNOS^Ser1177^). Total β-actin levels were used as loading control. A representative set of immunoblots is shown for experiments that were repeated independently 3 times. Each bar represents the mean ± SEM of densitometric analysis for phosphorylated proteins normalized to their respective total forms. *p < 0.05 *vs* respective basal.

Taken together our functional studies suggest that: 1. for concentrations higher than 1 μM, R-568 is able to produce a vasodilatant effect that does not differ between WKY and SHR rats, it is unlikely mediated by CaSR activation, and does not depend on NO production and/or other endothelial factors; 2. conversely, in the lower range (10 nM -1 μM) of concentrations, both endothelium and CaSR are required for R-568-dependent vascular activities, which translate in vasodilation in WKY and vasoconstriction in SHR, respectively. Based on the different consequences of eNOS activation under healthy and pathological conditions discussed above, we evaluated the ability of R-568 to increase phosphorylation (activation) of eNOS in the whole MVB homogenates. Moreover, since phosphorylation of eNOS by the upstream kinase Akt is an important mechanism regulating activation of eNOS in response to several ligands, the phosphorylation levels of eNOS and Akt were concomitantly measured in both WKY and SHR preparations. As shown in Fig 4 levels of phospho-eNOS and phospho-Akt increased proportionally to concentrations of R-568 administered, with a quantifiable effect on Akt starting at 100 nM (p < 0.05 *vs* respective unstimulated conditions). Thus, in normotensive WKY, R-568-mediated vasodilation was associated with an Akt-associated activation of eNOS. These findings corroborate literature data demonstrating that stimulation of CaSR induces eNOS activation and NO release, which in turn produces relaxation by regulating extracellular Ca^2+^ in mesenteric arteries from mice [19], and involves the heteromeric transient receptors TRPV4-TRPC1 channels in mesenteric arteries from rabbits [14].

Conversely, in preparations from SHR, no significant increase in phosphorylated levels of Akt and eNOS was detected in MVB stimulated with the same R-568 concentrations (Fig 4), suggesting the impairment of Akt/eNOS signaling activation in response to R-568. Therefore, differences in molecular mechanisms activated by R-568 in whole vessels from WKY and SHR support the diverging functional effects observed in these two strains.

### CaSR expression in WKY- and SHR-vSMCs

Endothelial cells constitute only a very small fraction of the total protein in a whole vessel. This, together with the concept that the phenotypic modulation of VSMCs is known to play a pivotal role in hypertension-induced vascular changes [51], advocate for our subsequent studies investigating the role of vSMCs on R-568-mediated effects. The presence of CaSR on vSMCs is still controversial [49, 50, 52, 53], with some studies reporting the expression of CaSR in human and animal models [5, 6, 48, 49] while some others have failed to detect the cognate CaSR in vSMCs [52]. Furthermore, the discovery and cloning of a novel human family of C GPCR, with a significant homology to the human CaSR, suggest the presence of a distinct calcium sensing receptor termed GPRC6A and its possible activation by high concentrations of extracellular calcium and calcimimetics [54, 55].

**Fig 5:**
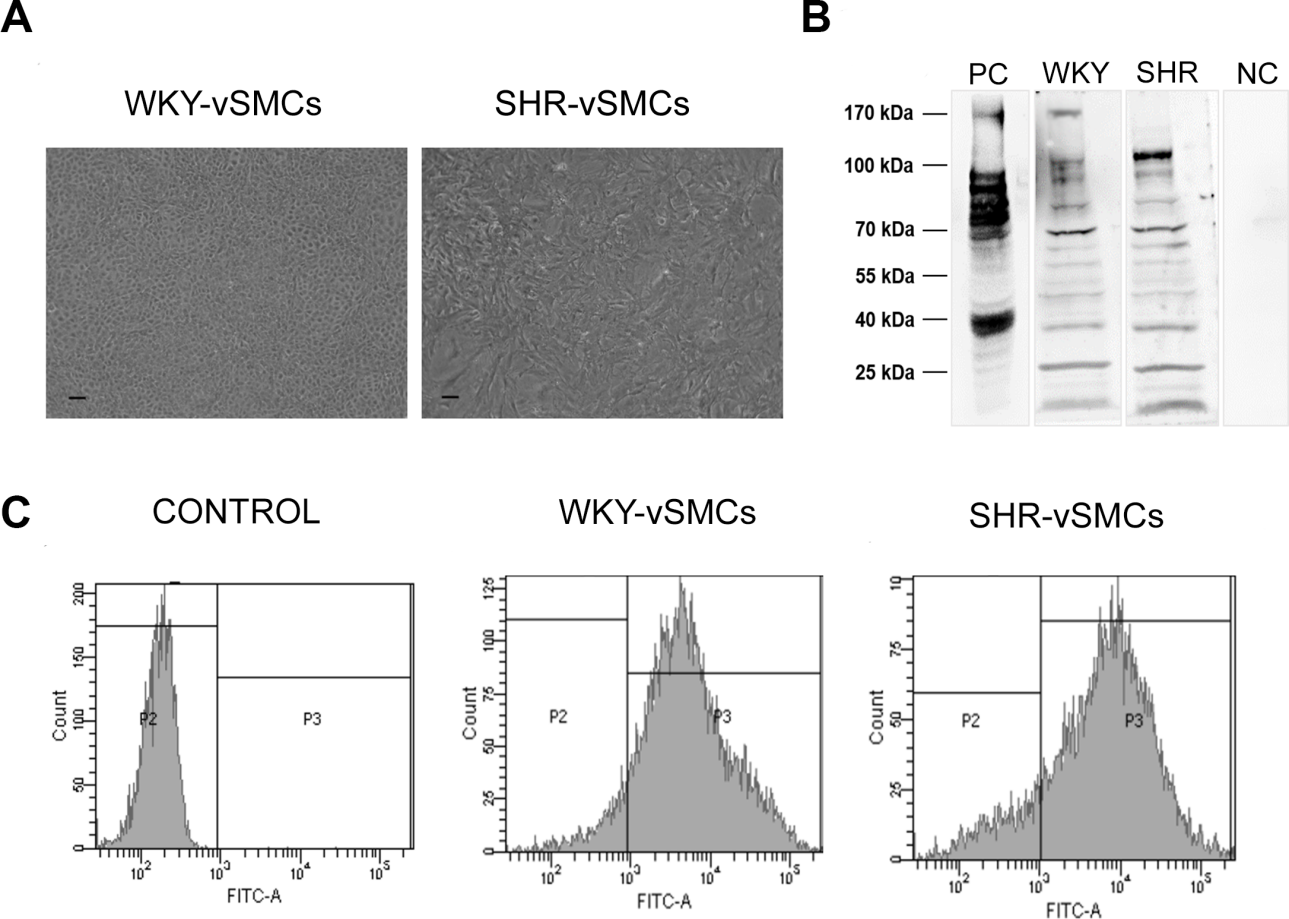
CaSR expression in WKY- and SHR-vSMVCs. (A) Representative morphometric aspect of confluent vSMC cultures from normotensive rats (WKY) and spontaneously hypertensive rats (SHR) (magnification x 10; scale bar: 300 μM). The cells were plated and morphologically examined three different times in the same experimental conditions. (B) Representative pattern of electrophoretic bands (170–100, 70–55 and 40–25 kDa) that results from immunoblotting analysis of CaSR expression in WKY- and SHR-vSMCs lysates and its negative (NC, HEK293 empty vector transfected control cell lysate) and positive (PC, HEK293 CaSR transiently transfected cell lysate) controls. (C) Representative images of α-SMA flow cytometry analysis in WKY-and SHR-vSMCs and secondary antibody alone (control).

Here, following the isolation of vSMCs from the thoracic aorta of WKY and SHR rats, we first confirmed their phenotype by evaluating the contractile marker α-SMA that resulted ≥ 90% for both vSMCs strains (Fig 5C). Then, we compared the morphological characteristics of cell cultures from WKY and SHR. As shown in Fig 5A, the WKY-vSMCs showed a ‘‘hill and valley” morphology typically observed in cultured vSMCs [56]. SHR-vSMCs, on the contrary, showed an atypical morphology, suggestive of altered mechanisms controlling contact inhibition. To ascertain if changes in vSMCs morphology might also reflect a differential expression of CaSR protein between WKY- and SHR-vSMCs, a western blot analysis was performed in cell lysates under reducing conditions. As shown in Fig 5B, and consistent with our and other previous reports on HUVECs [17, 57] we observed a pattern of electrophoretic bands (170–100, 70–55 and 40–25 kDa) indicative of CaSR protein. The different molecular weight is depending upon the extent of proteins glycosylation and the presence of its monomeric or multimeric form [58, 59].

On this respect, it is important to specify that the functional CaSR resides on the cell surface in the dimeric form, while the intracellular pool of CaSR has been considered a rapidly mobilizable “storage form” [60, 61] with a potential role in the modulation of intracellular activities triggered by changes in endoplasmic reticulum Ca^**2+**^ and/or glutathione [61].

Interestingly, this pattern of bands reveal the mature and biologically active dimeric form of the receptor (170 kDa) in the WKY-vSMCs, whereas in the SHR-vSMCs the monomeric CaSR protein (70 kDa) resulted the most expressed (Fig 5B).

### Effect of R-568 on Akt activation and NO production in vSMCs from WKY and SHR rats

**Fig 6:**
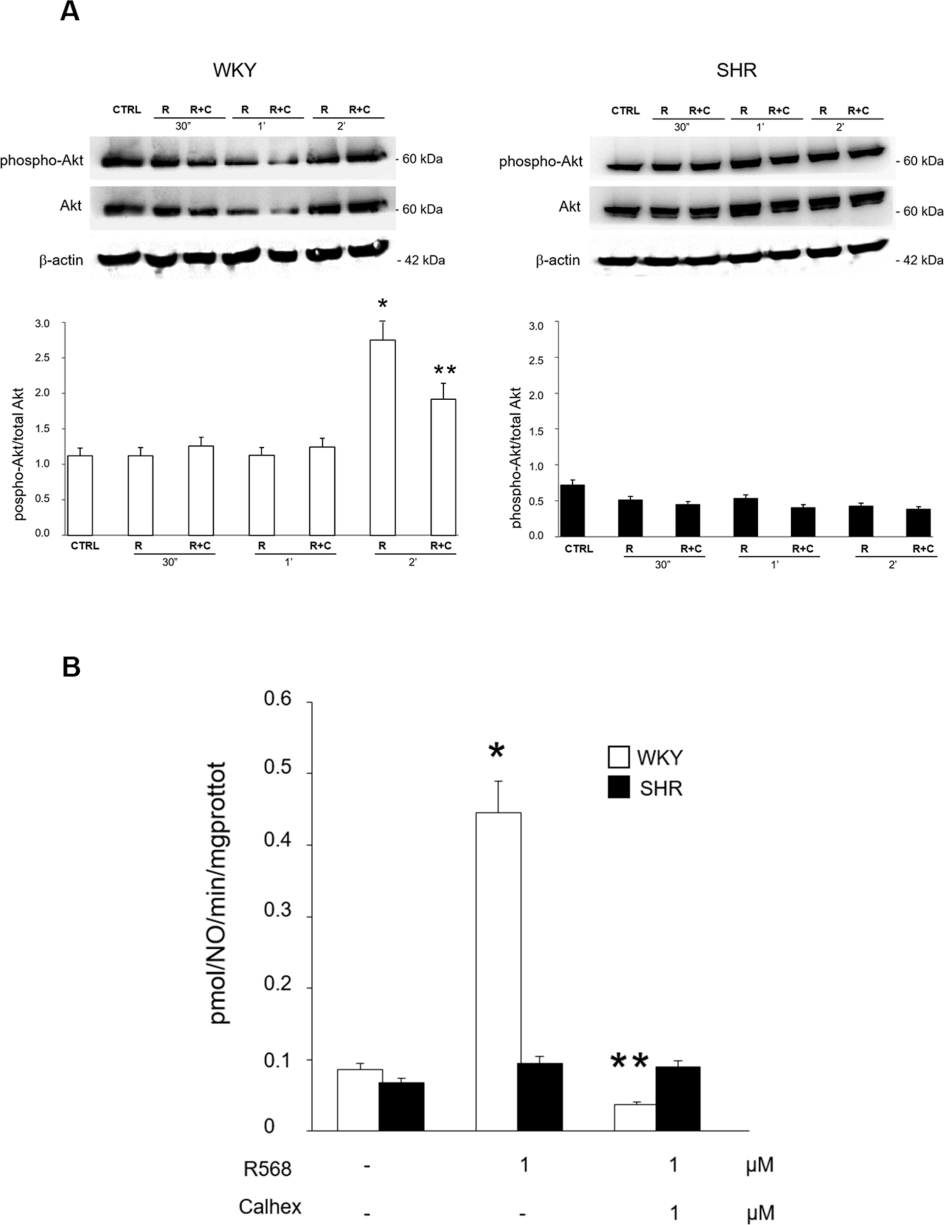
Effect of R-568 on Akt activation and NO production in vSMCs from WKY and SHR rats. (A) vSMCs stimulated with 1μM of R568 (R) at different times (30 seconds, 1 minute, 2 minutes) in the presence or absence of Calhex 231 (C, 1 μM) were subjected to immunoblotting for total and phosphorylated forms of Akt (phospho-Akt^Ser473^). Total β-actin levels were used as loading control. A representative set of immunoblots is shown for experiments that were repeated independently 3 times. Each bar represents the mean ± SD of densitometric analysis for phosphorylated proteins normalized to their respective total forms. *p < 0.02 *vs* respective basal (CTRL), **p < 0.05 R+C *vs* respective R. (B) NO production determined by conversion of L-[3H]-arginine into L-[3H]-citrulline in vSMCs stimulated with 1μM of R568 in the presence or absence of Calhex 231 (1 μM), data are expressed as pmol/NO/min/mgprottot (picomoles /Nitric Oxide/ minutes/ milligrams protein total). Each bar represents the mean ± SD of 3 independent experiments. *p < 0.02 R568 *vs* respective basal, **p < 0.01 R568+Calhex *vs* respective R568.

The possibility that, besides endothelium, CaSR expressed on vSMCs may also participate to vascular activities of R-568 was then evaluated. As shown in Fig 6A, after two minutes of stimulation, R-568 (1 μM) induced a significant increase in Akt phosphorylation levels (p < 0.02 *vs* respective unstimulated conditions) on WKY-vSMCs while no significant activation of Akt signaling was found on SHR-vSMCs. Pre-incubation with CaSR inhibitor Calhex (1 μM) completely reverted the R-568-dependent phosphorylation of Akt in WKY-vSMCs (p < 0.05 *vs* respective R-568 alone).

Similar to endothelial cells, activation of Akt in vSMCs has been linked to activation of eNOS, whose expression has previously been unequivocally documented in vSMCs from mice [62] as well as from humans, under both basal and pathological conditions [63, 64]. Thus, NOS activity in response to R-568 was evaluated in WKY- and SHR-vSMCs by a conventional method indirectly measuring the conversion of the substrate L-[3H]-arginine into L-[3H]-citrulline [28, 31]. As shown in Fig 6B, vSMCs stimulation with R-568 (1 μM) was associated to a significant production of NO in WKY cells (p < 0.02 *vs* respective unstimulated conditions) that reverted to baseline levels when cells were concomitantly treated with the CaSR inhibitor Calhex 231 (1 μM) (p < 0.01 *vs* respective R-568 alone).

Similar to Akt phosphorylation levels, no significant increase in NO production was measured in vSMCs from SHR. Collectively, these findings support the hypothesis that the absence of the CaSR active dimeric form in vSMCs from SHR rats may contribute to explain the impaired vasorelaxant effect observed in response to R-568 in MVB from SHR, with respect to WKY [33].

## Conclusions

Taken together, these findings highlight new mechanisms of action of calcimimetics in modulating vascular tone, both in normal and pathological conditions such as hypertension. In isolated vessels from hypertensive compared to normotensive rats, the calcimimetic R-568 exerts different dose-dependent vasodilatory effects. Particularly, at the lowest R-568 concentrations used, its effect in isolated vessels results opposite: vasodilatory in WKY and vasoconstrictor in SHR. In both cases dependent on the CaSR and the endothelium itself, while the involvement of vSMCs in the R-568 CaSR-dependent vasodilatation might be assumed only for WKY rats.

Although further studies are necessary to thoroughly understand these effects, our study suggest that calcimimetics might be useful to regulate vascular tone and this could represent the beginning of a new therapeutic approach.
